# Characterization of transcription factor genes related to cold tolerance in *Brassica napus*

**DOI:** 10.5808/gi.21055

**Published:** 2021-12-31

**Authors:** Mayur Mukut Murlidhar Sharma, Rahul Vasudeo Ramekar, Nam-Il Park, Ik-Young Choi, Seon-Kang Choi, Kyong-Cheul Park

**Affiliations:** 1 Department of Agriculture and Life Industries, Kangwon National University, Chuncheon 24341, Korea; 2 Department of Plant Science, Gangneung-Wonju National University, Gangneung 25457, Korea

**Keywords:** *Brassica napus*, cold-stress, cold-tolerance, tanscription factors

## Abstract

*Brassica napus* is the third most important oilseed crop in the world; however, in Korea, it is greatly affected by cold stress, limiting seed growth and production. Plants have developed specific stress responses that are generally divided into three categories: cold-stress signaling, transcriptional/post-transcriptional regulation, and stress-response mechanisms. Large numbers of functional and regulatory proteins are involved in these processes when triggered by cold stress. Here, our objective was to investigate the different genetic factors involved in the cold-stress responses of *B. napus*. Consequently, we treated the Korean *B. napus* cultivar Naehan at the 4-week stage in cold chambers under different conditions, and RNA and cDNA were obtained. An *in silico* analysis included 80 cold-responsive genes downloaded from the National Center for Biotechnology Information (NCBI) database. Expression levels were assessed by reverse transcription polymerase chain reaction, and 14 cold-triggered genes were identified under cold-stress conditions. The most significant genes encoded zinc-finger proteins (33.7%), followed by MYB transcription factors (7.5%). In the future, we will select genes appropriate for improving the cold tolerance of *B. napus*.

## Introduction

*Brassica napus* (genome AnAnCnCn) was formed recently by allopolyploidy between ancestors of *Brassica oleracea* (Mediterranean cabbage, genome CoCo) and *Brassica rapa* (Asian cabbage or turnip, genome ArAr) and is polyphyletic [1,2]. In natural habitats, plants face stressful conditions, ranging from biotic stresses, including pathogens, insects and weed infestation, and abiotic stresses caused by fluctuations in environmental conditions, like temperature, humidity, and sunlight. Drought, salinity and freezing are major abiotic stresses that cause adverse effects on crop growth and productivity by disturbing the physio-chemical balance in plant cells. Plants modify themselves at the cellular level by regulating different pathways governed by several genes to minimize the harm caused by an elicitor and to protect from further damage [3,4]. Low temperature is a major abiotic stress affecting plant growth in South Korea, where the temperature falls below −15°C in winters. The cold conditions freeze intracellular water, resulting in its movement out of the cell, owing to the potential gradient, which leads to the rupturing of cells, causing prominent damage to plants in the form of leaf wilting and burning, as well as possible plant death [5]. The cold tolerance of a plant is induced in response to low non-freezing temperatures and involves cold acclimation, which requires complex regulatory mechanisms [6]. The cold-tolerance mechanism comprises diverse pathways that involve networks of multiple components, including proteins, such as heat-shock and antioxidative enzymes [7], phytohormones, such as ethylene and abscisic acid [8], and cold-responsive genes, which are indispensable for coding many necessary proteins involved in cellular responses [9]. These components can be manipulated to make the plant resilient against stressful environmental conditions. Despite considerable efforts, traditional breeding approaches have resulted in only modest improvements in abiotic stress tolerance because much of the required genetic variation has been lost during domestication and modern breeding-associated selection [10,11]. Additionally, abiotic stress resistance, as a polygenic trait, is governed by complex genetic networks that make it difficult for the breeders to converge the polygenes in a single plant system [12]. Another alternative to conventional breeding is marker-assisted selection, which uses molecular markers in association and linkage mapping. From the association or linkage maps, quantitative trait loci associated with agronomic traits may be identified and used for gene pyramiding or recurrent parent genome analyses [13–15]. Marker-assisted selection has been successfully exploited to confer cold-stress resistance in *B. napus* [16]. Recently, molecular, biological, and physiological studies investigating cellular responses in plants to abiotic stress have progressed significantly. The genetic factors behind these phenomena have been largely uncovered. The cloning and overexpression of these factors through biotechnological approaches, as well as genome editing, have resulted in conferring cold-resistance to plants [17,18]. Knowledge of the molecular basis of freezing tolerance will help develop tools that can effectively increasing the freezing tolerance of plants. Recently, several different gene transfer approaches to improve plant-stress tolerance, such as tissue electroporation [19], microinjection [20] and particle bombardment [21], have been employed. The transferred genes included those encoding enzymes required for the biosynthesis of various osmoprotectants that are responsible for protecting plant cells from damage under stress conditions [22].

When a plant is subjected to abiotic stresses, an assortment of genes having diverse functions are induced, or repressed, to synthesize key proteins and enzymes to protect the plant. These proteins may be categorized into two groups. The first group includes functional proteins, such as late embryogenesis abundant proteins, antifreeze proteins, molecular chaperones, key enzymes for osmolyte biosynthesis, like proline, sugar and sugar alcohols, betaines, detoxification enzymes, water-channel proteins and membrane transporters, which are directly associated with protecting plants from the ill effects of abiotic stresses. The second group includes regulatory proteins that control signal transduction and stress-responsive gene expression. These include various transcription factors, protein kinases, enzymes involved in phospholipid metabolism, and other signaling molecules, such as calmodulin-binding protein and 14-3-3 protein [23]. Analyzing and elucidating the functions of these genes are critical for further understanding the molecular mechanisms governing plant abiotic stress tolerance and may help in the genetic manipulation of crops for enhanced stress tolerance [24]. Previous studies have focused on targeting both the regulatory and functional genes involved in cold-stress tolerance [25,26]. Among these genes, protein kinases [27] heat-shock proteins [28], late embryogenesis abundant proteins [29] and genes encoding transcription factors appear to be most useful in improving stress tolerance in plants [23].

Transcription factors act as trans-acting elements through their specific binding to cis-acting elements in the promoters of target genes, and this allows them to play central roles in regulating the expression of downstream genes [30]. Therefore, the study of different genes having regulatory and functional roles is important for understanding the mechanisms involved in regulating stress responses as well as to fully elucidate the mechanisms of tolerance against abiotic stress. [31]. The objectives of this study in *B. napus* were as follows: () to perform a comprehensive analysis of cold-related genetic factors; and (2) to form a shortlist of suitable candidate genetic factors that can be used for enhancing cold tolerance. The workflow of this study is depicted in Supplementary Fig. 1.

## Methods

### Plant material, growth conditions and cold treatment.

A cold-tolerant cultivar, Naehan, was used in our study. *Brassica napus* seeds were sterilized, sown on Murashige-Skoog medium and incubated in an illuminated incubation chamber with a 16-h/8-h (light/dark) photoperiod, a photonflux density of 200 μmol m^-2^s^-1^ and a relative humidity of 70% at 25°C for 7 days. The seedlings were then planted in soil and grown at 25/22°C (day/night) with a 16-h/8-h (light/dark) photoperiod, a photonflux density range of 500–600 μmol m^-2^ s^-1^ and a relative humidity range of 60%–70% in a greenhouse. To investigate the cold-induced regulatory proteins, plants at the three-leaf stage were given different cold treatments, 10, 6, 3, 0, −3 and −6°C, for 8 h and randomly selected for sampling. A room temperature treatment served as the control.

### RNA isolation and cDNA synthesis

Leaf tissue was placed in a mortar containing liquid nitrogen and homogenized with a pestle. Approximately 100 mg of the powder was transferred to a 1.5-µL microtube. Total RNA was isolated using a Ribospin Plant RNA isolation kit (Geneall Biotechnology Co. Ltd., Seoul, Korea) as per the protocol provided by the manufacturer. RNA integrity was determined by the RNA Integrity Number, which was calculated using an Agilent 2100 Bioanalyzer (Agilent Technologies Korea Ltd., Seoul, Korea). The samples with RNA Integrity Number values greater than eight were selected for further analysis. Using total RNA as the template, cDNAs were synthesized using Geneall 2X HyperScript reverse transcription polymerase chain reaction (RT-PCR) master mix with oligo dT (Geneall Biotechnology Co. Ltd.) as per the protocol provided by the manufacturer. Reaction details for the RT-PCR are as follows: Preheating of total RNA at 65°C for 5 min followed by heating the reaction mixture to 55°C for 60 min and 95°C for 5 min.

### *In silico* analysis and primer designing

The nucleotide sequences of 80 cold-related genes were downloaded from the NCBI database (https://www.ncbi.nlm.nih.gov). These genes encoded different necessary proteins. Gene-specific primers were designed for the 80 genes using Primer 3 software. (https://bioinfo.ut.ee/primer3-0.4.0/).

### Validation of gene-specific primers

Here, the *B. napus* actin gene was used as an internal reference. Gene-specific primers were validated by performing general PCR using cDNA as the template. The PCR reaction components were 10× buffer, dNTPs (0.5 mm), forward primer (10 pm), reverse primer (10 pm), DNA template (20 ng), and Taq polymerase (0.025 units). The PCR reaction was as follows: 95°C for 10 min and 40 cycles of 95°C for 5 s, 56°C for 15 s and 72°C for 30 s. The PCR products were screened using agarose gel electrophoresis.

## Results

### Data extraction and *in silico* identification of annotated cold-related genes

**Fig. 1.**
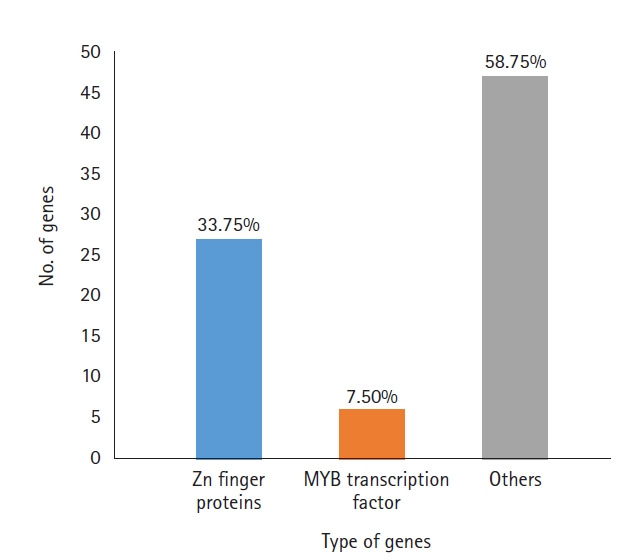
Numbers of cold-responsive genes belonging to different families in our data.

A total of 919 functionally annotated cold-related genes, including both regulatory and functional genes belonging to diverse families, were retrieved from the NCBI database. Among these genes, those having regulatory functions were identified and listed according to their families. A total of 80 genes were found (Supplementary Table 1) with zinc-finger protein genes making up the highest percentage (33.7%), followed by myeloblastosis transcription factors (MYB) (7.5%). Other genes included TCP transcription factors, basic leucine zipper domain-containing transcription factors and those encoding stress-related proteins (58.7%) (Fig. 1). Among the zinc-finger proteins, seven contained the C3HC4 domain and three each contained C2H2 or the B-box. Other members, such as WRKY, GATA, GRAS, NAC, and E2F/dimerization partner A were each represented by a solitary member in our data.

### Expression profiling of genes by RT-PCR

To assess the mRNA transcript levels of the cold-responsive transcription factor genes, RT-PCR was performed using an endogenous gene (actin) as an internal standard for 80 genes (Supplementary Fig. 2). On the basis of gel-electrophoresis results, the proper amplified genes were sorted for the selection of candidate genes. The genes were sorted by band intensity, followed by RT-PCR. In total, 14 genes (Table 1) showing differential gene expression levels corresponding to the decrease in temperature were selected. The expression levels of group members were high as compared to the other candidate genes after the temperature decrease.

### Assortment of candidate genes

**Fig. 2.**
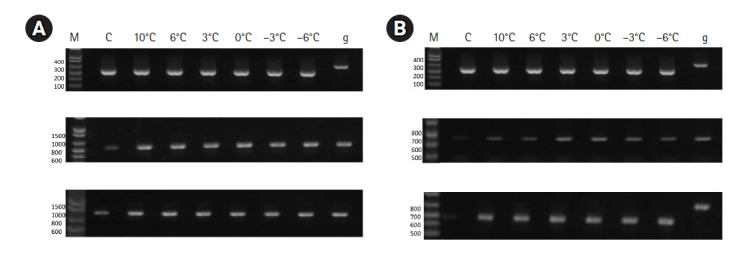
Expression levels of cold-responsive zinc finger protein genes and MYB transcription factor genes at control and lower temperatures as assessed by reverse transcription polymerase chain reaction in *Brassica napus* cv. Naehan subjected to low temperatures. (A) Zinc-finger genes. Reference gene actin (upper), EV199365 (zinc-finger family protein) (middle), EV196553 (zinc-finger family protein) (lower). (B) MYB transcription factors. Reference gene actin (upper), EV190420 (MYB transcription factor) (middle), EV196127 (MYB transcription factor) (lower).

The total sorted 14 genes encoded diverse proteins, including zinc fingers (8), MYB transcription factors (2), membrane protein (1), KANADI transcription factor (1), stress-related protein (1), and dimerization partner A transcription factor (1). Details on the primers for these 14 genes are described in Table 2. The first category includes the genes encoding zinc-finger proteins, including EV189678 (zinc-finger [MYND type] family protein), EV192654 (zinc-finger protein), EV192647 (zinc-finger [B-box type] family protein), CB686176 (zinc-finger [AN1-like] family protein), EV198845 (zinc-finger [B-box type] family protein), and EV197045 (zinc-finger family protein). The proteins EV199365 and EV196553, encoded by two additional zinc-finger genes, showed weak expression levels at the control temperature compared with at lower temperatures, as shown in Fig. 2A relative to the expression of the actin housekeeping gene. The second category of promising regulatory genes includes MYB transcription factors (Fig. 2B). EV190420 and EV196127 (MYB family transcription factor), and the expression levels of the genes encoding these proteins increased as the temperature decreased.

## Discussion

Transcription factors are necessary elements for conducting the central dogma of life, and they affect the transcription levels of genes [32]. In total, 8%–10% of a plant genome is translated into transcription factors [33]. Information on regulatory proteins provides us the opportunity to manipulate plant responses to external factors at the regulatory level, such as improving cold tolerance in *B. napus*. The increased availability of proper cold-responsive regulatory protein–encoding genes provides us with the more options and opportunities to investigate those that may be candidates for improving cold tolerance in *B. napus*. Among them, the dehydration-responsive element-binding protein (DREB) transcription factors from the ethylene response factor family have gained much attention owing to their involvement in the regulation of many stress-related genes that play important roles in the cascading responses to environmental stimuli [34]. They have significant roles in the tolerance to cold stress in *B. napus* [35]. Here, different regulatory factors belonging to different families related to cold-stress response were identified. Among the transcription factors, the MYB members were the most abundant. Different members of the MYB transcription factor family also bind to specific cis sites of cold-responsive genes to induce their expression, and they help stabilize plant cells under stress conditions through a complex of cold tolerance- and cold acclimation-related pathways. MYB transcription factor family members are actively involved in important regulatory roles in responses, and tolerance levels, to other abiotic stresses, such as salinity [13] and drought [13]. The widespread members of the MYB family have diverse roles in multiple pathways that are indispensable to plant growth and development and to plant adaptation to various environmental conditions. Our study investigated MYB transcription factors, many of which did not show expression at the control temperature but increased increasing their expression level as the temperature decreased. An R2R3-MYB protein has been reported as being involved in the biosynthesis of proanthocyanidin, which is required for seed coloring in *B. napus* [36]. Glucosinolate (GSL) is a well-known secondary metabolite in the Brassicacea family, and its synthesis in *Arabidopsis thaliana* is governed by a member of MYB transcription factor family. However, owing to the complexity of the genome, GSL biosynthesis has not been studied extensively in other *Brassica* species [37]. Few genome-wide association and transcriptome analyses in B .napus have shown MYB transcription factor roles in modulating reactive oxygen species [38] and regulating salt-stress tolerance [39]. Here, the largest number of transcription factors belonged to the zinc-finger family, and these members play multiple roles in plant growth and development. In our study, the identified 14 cold-responsive factor genes from different families contained mainly zinc-finger proteins, followed by MYB transcription factors. These genes had regulatory roles in the several pathways responsible for cold acclimation, as well as in protecting the plant cell against cold stress by conferring cold tolerance. Zinc-finger transcription factors are crucial in many plant regulatory pathways as well as in pathways involved in tolerance to various abiotic stresses. In *Arabidopsis*, the regulation of cadmium tolerance by a zinc finger of *A. thaliana* 6 (*ZAT6*) has been reported [40]. In a study conducted by Kim et al. [41], a zinc-finger transcription factor *Capsicum annuum* zinc finger protein 1 (*CAZFP1*) was found to be induced in defense against pathogens and under drought-stress conditions. *Brassica rapa* Ring zinc finger protein 1 (*BrRZFP1*) is involved in responses to multiple abiotic stresses in *B. rapa* [42]. The overexpression of zinc-finger genes, such as *Oryza sativa indica* stress-associated protein 8 (*OsiSAP8*) [43], *O. sativa* cold-inducible (*OsCOIN*) [44], *O. sativa* dehydration-responsive element-binding protein 1 (*OsDREB1*) [30], reactive oxygen species-basic leucine zipper (ROS-bZIP) [45], *SNAC2* [46], and *OsNAC6* [47], confer cold-stress tolerance at the seedling stage in rice. The members of the zinc-finger family are involved in different regulatory roles in the life processes of different plants. In *B. napus*, a particular classes of zinc-finger protein‒encoding genes having B-box, AN1 and MYND domains were found. The AN1 domain-containing zinc-finger proteins are involved in immune responses in humans [48], and recently, they have been found to play roles in combating multiple abiotic stresses in plants [49]. In a genome-wide identification conducted by [50] in *B. napus*, the AN1 proteins were induced by different kinds of stress, including cold stress. Another class of zinc-finger proteins is the B-box proteins. B-box proteins play multiple roles in different plants. In *Arabidopsis*, they are found in abundance and linked to circadian-associated events [51]. In apples, a B-box protein, *Malus domestica* B-box 20 (MdBBX20) is involved in anthocyanin accumulation in the epicarp [52]. Their involvement in the regulation of abiotic stresses has been determined. In *Arabidopsis*, they are induced in response to a cold environment and enhance the plant’s tolerance to cold conditions [53]. The role of the gene *B. napus* cold-regulated 25 (BnCOR25) in conferring cold tolerance to *B. napus* has been confirmed by its overexpression in *Arabidopsis* and yeast [54].

## Figures and Tables

**Table 1.** Identified candidate cold-responsive genes based on RT-PCR

Gene category	No. of genes
Zinc-finger protein	8
MYB transcription factor	2
Membrane protein	1
KANADI transcription factor	1
Stress-related protein	1
Dimerization partner A transcription factor	1
Total	14

RT-PCR, reverse transcription polymerase chain reaction.

**Table 2.** Primer details for 14 *Brassica napus* candidate cold-responsive genes

No.	Gene ID	Gene description	Primer sequence (F & R)	Primer length	Tm (℃)
1	EV189678	Zinc finger protein	F: GATCACATGCAAGAGACTGAAT	22	51
			R: ATTTGAGAGAGAGGTAAGAGCG	22	53
2	EV192647	Zinc finger protein	F: GTCCGAAAGTTTGGCTAATG	20	50
			R: GTTCCCACAGATTCTTTTAAGC	22	51
3	EV197045	Zinc finger protein	F: CATCAACGGTAGATCCAGATG	21	52
			R: TCATACTGTGGGTCTCTGTCTC	22	55
4	EV192654	Zinc finger protein	F: TTAGTTCACCGAGATTAGCTTCG	23	60
			R: GAGAACTCTGGTATCGGAGGAAT	23	60
5	CB686176	Zinc finger protein	F: TGAATCTCTGCTCCAACTGCTA	22	60
			R: GGTGAATGAAAACTGTCTCAACC	23	59
6	EV198845	Zinc finger protein	F: AATGTTCTGTGAGTCAGACCAG	20	60
			R: TCATCATCGGAGTTAAGTTCTG	20	60
7	EV199365	Zinc finger protein	F: ATTGCACTCAATTTGTTAGCC	22	60
			R: TGCCATATCCACAAGTCATAAT	23	60
8	EV196553	Zinc finger protein	F: CTTACGTGATTGTGAGTTTTCC	22	53
			R: GTTAGAGGCGGTAAGAGAAGAG	22	51
9	EV190420	MYB trancription factor	F: TCTCTCTCTCTCTCTCTTCGCT	20	52
			R: TTCTTAGTGTTCCCTCCTTCAT	22	53
10	EV196127	MYB trancription factor	F: ACACTTCTCCAGCTACGTCCA	21	60
			R: TGTCTCTCAGGACTCCAGCAT	21	60
11	EV198916	Stress-related protein	F: AAATTGATCACCGAGTTCTTCT	22	49
			R: AAGCAGAATAATGGAACAAGGT	22	49
12	EV203394	Membrane protein	F: ACTTTTAGATTTTGAGCGGTCT	22	49
			R: CAATGACCATAAAAGCTATGGA	22	49
13	EV190408	KANADI	F: AATGGTAGCTGCGGTACG	18	50
			R: TGATTGTGGTGATTAGGGTAAA	22	49
14	EV200969	DPA transcription factor	F: GAGAGAAGAGAGGATGGAGATG	22	55
			R: GCATGAGGATTTGTCTCAAGTA	22	51

## Supplementary Materials

Supplementary Table 1. Total numbers of cold-related genes identified in our data

Supplementary Fig. 1. A representation of the workflow covering the hierarchical information in this study.

Supplementary Fig. 2. Expression profiling of all the candidate cold-related genes identified in our data.
