# Supplementary material for: Characterization of transcription factor genes related to cold tolerance in Brassica napus
Source: Genomics Inform. 2021 Dec 31;19(4):e45. doi: 10.5808/gi.21055 (PMC8752983; doi:10.5808/gi.21055)
Supplement: Supplementary Table 1. — Total numbers of cold-related genes identified in our data [file gi-21055suppl1.pdf]

Supplementary materials

Supplementary Table 1. Total numbers of cold-related genes identified in our data

Serial No.	GenBank ID	Gene family	No. of genes
1	EV201738	Putative transcription factor [*Arabidopsis thaliana*]	1
2	BQ704445	Transcription factor IIIA	1
3	EV196426	AP2 domain-containing Transcription factor	1
4	EV199892	bZIP	2
5	EV202190		
6	EV193738	Putative transcription factor	2
8	EV190626		
9	EV201258	BLH3	1
10	EV196127	MYB	5
11	EV195251		
13	EV201981		
14	EV197426		
15	EV190223		
16	EV199120	Putative CCAAT-binding transcription factor	1
17	EV198052	NAC	1
18	EV200969	DPA	1
19	EV200522	Homeobox protein	1
20	EV190408	KANADI	1
21	EV202135	TCP	3
22	EV196781		
23	EV202588		
24	EV195578	HD-ZIP	1

25	EV198388	GATA	1
26	EV199212	GRAS (Scarecrow-like transcription factor PAT1)	1
27	EV191966	E2F	1
28	EV193324	WRKY	1
29	CB686269	ZINC finger proteins	27
30	BQ705034		
31	EV198837		
32	EV197840		
33	EV198570		
34	CB686176		
35	EV199810		
36	EV189678		
37	EV201626		
38	EV193938		
39	EV197045		
40	EV200021		
41	EV199795		
42	EV195398		
43	EV202561		
44	EV200607		
45	EV197345		
46	EV197756		
47	EV190887		
48	EV190516		
49	EV194443		
50	EV193913		
51	EV192647		
52	EV198665		
53	EV198845		

54	EV198940		
55	BQ704703	SERUM GLYCOPROTEIN	1
56	EV190774	Fatty acid elongase	3
57	EV199497		
58	EV189416		
59	EV201838	BRASSINOSTEROID INSENSITIVE 1	1
60	EV198882	MBOAT	1
61	EV190084	LrgB-like	1
62	EV196663	Peroxin-13 family	1
63	EV198661	Membrane protein	1
64	EV202828	RCK N-terminal	1
65	EV194405	Protein kinase	1
66	EV194379	Peptidase_M1	1
67	EV196292	ATPase	1
68	EV203394	Integral membrane HPP family protein	1
69	BQ704177	Stress responsive protein	1
70	EV198916	Stress protein	1
71	CB686236	Heat shock protein	8
72	EV196085		
73	EV192477		
74	EV203319		
75	EV195550		
76	EV196670		
77	EV193407		
78	EV191077		
79	EV189243	Transposon	2
80	EV199651	Transposon	
