# Supplementary material for: Characterization of transcription factor genes related to cold tolerance in Brassica napus
Source: Genomics Inform. 2021 Dec 31;19(4):e45. doi: 10.5808/gi.21055 (PMC8752983; doi:10.5808/gi.21055)
Supplement: Supplementary Fig. 1. — A representation of the workflow covering the hierarchical information in this study. [file gi-21055suppl2.pdf]

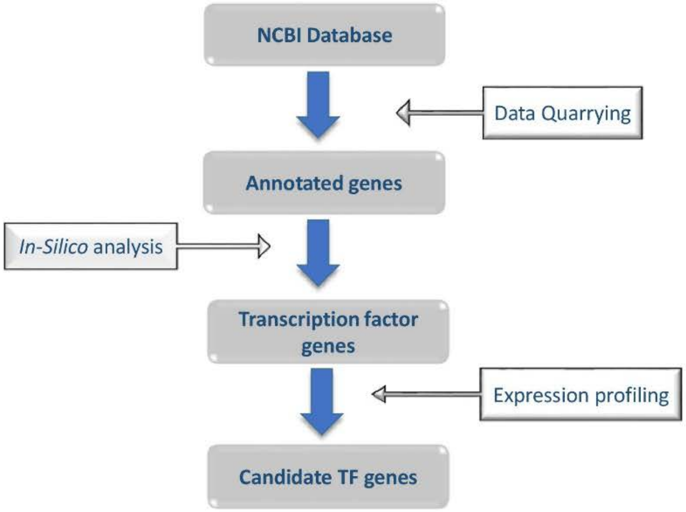

Supplementary Fig. 1. A representation of the workflow covering the hierarchical information in this study.
