# Supplementary material for: Characterization of transcription factor genes related to cold tolerance in Brassica napus
Source: Genomics Inform. 2021 Dec 31;19(4):e45. doi: 10.5808/gi.21055 (PMC8752983; doi:10.5808/gi.21055)
Supplement: Supplementary Fig. 2. — Expression profiling of all the candidate cold-related genes identified in our data. [file gi-21055suppl3.pdf]

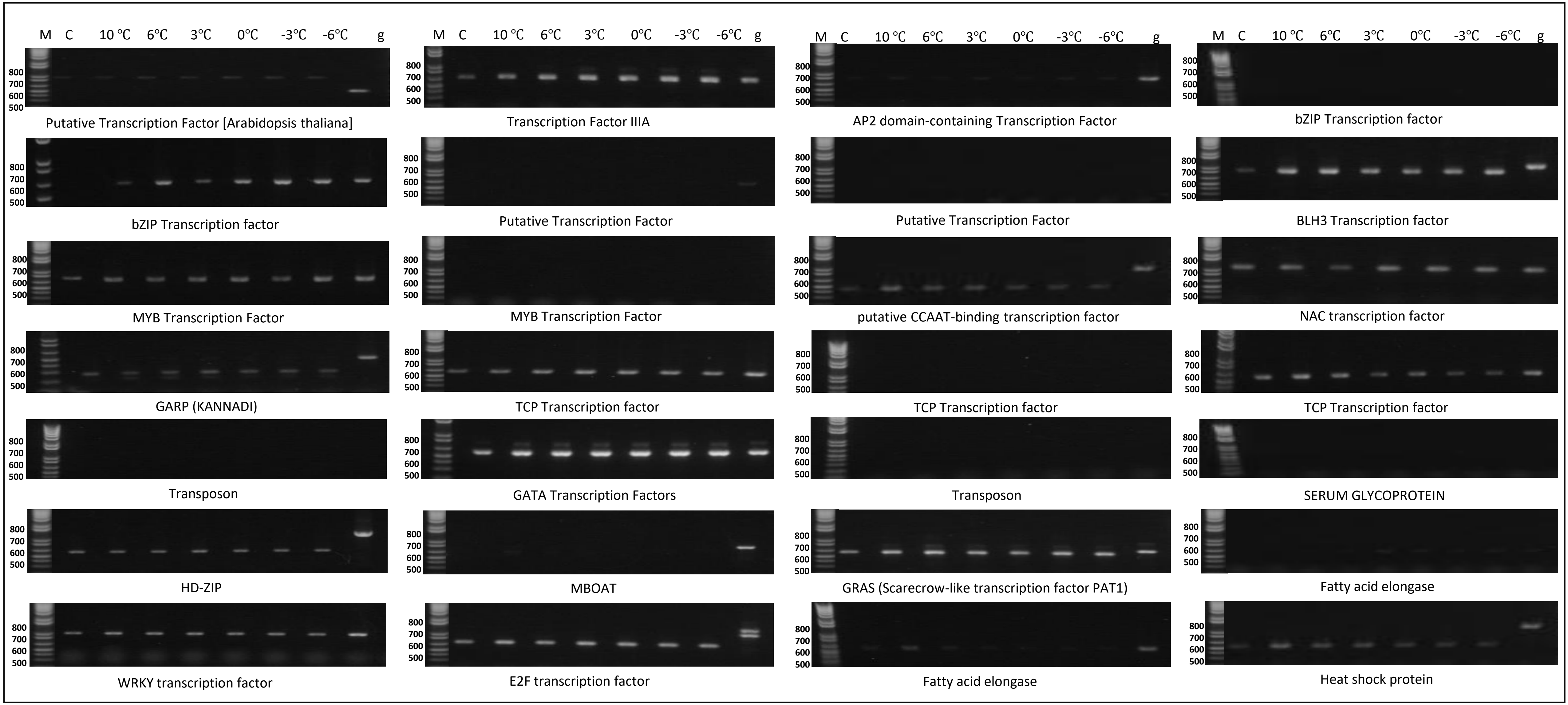
M C 10 °C 6°C 3°C 0°C -3°C -6°C g
800
700
600
500
Putative Transcription Factor [Arabidopsis thaliana]
Transcription Factor IIIA
AP2 domain-containing Transcription Factor
bZIP Transcription factor
bZIP Transcription factor
Putative Transcription Factor
Putative Transcription Factor
BLH3 Transcription factor
MYB Transcription Factor
MYB Transcription Factor
putative CCAAT-binding transcription factor
NAC transcription factor
GARP (KANNADI)
TCP Transcription factor
TCP Transcription factor
TCP Transcription factor
Transposon
GATA Transcription Factors
Transposon
SERUM GLYCOPROTEIN
HD-ZIP
MBOAT
GRAS (Scarecrow-like transcription factor PAT1)
Fatty acid elongase
WRKY transcription factor
E2F transcription factor
Fatty acid elongase
Heat shock protein

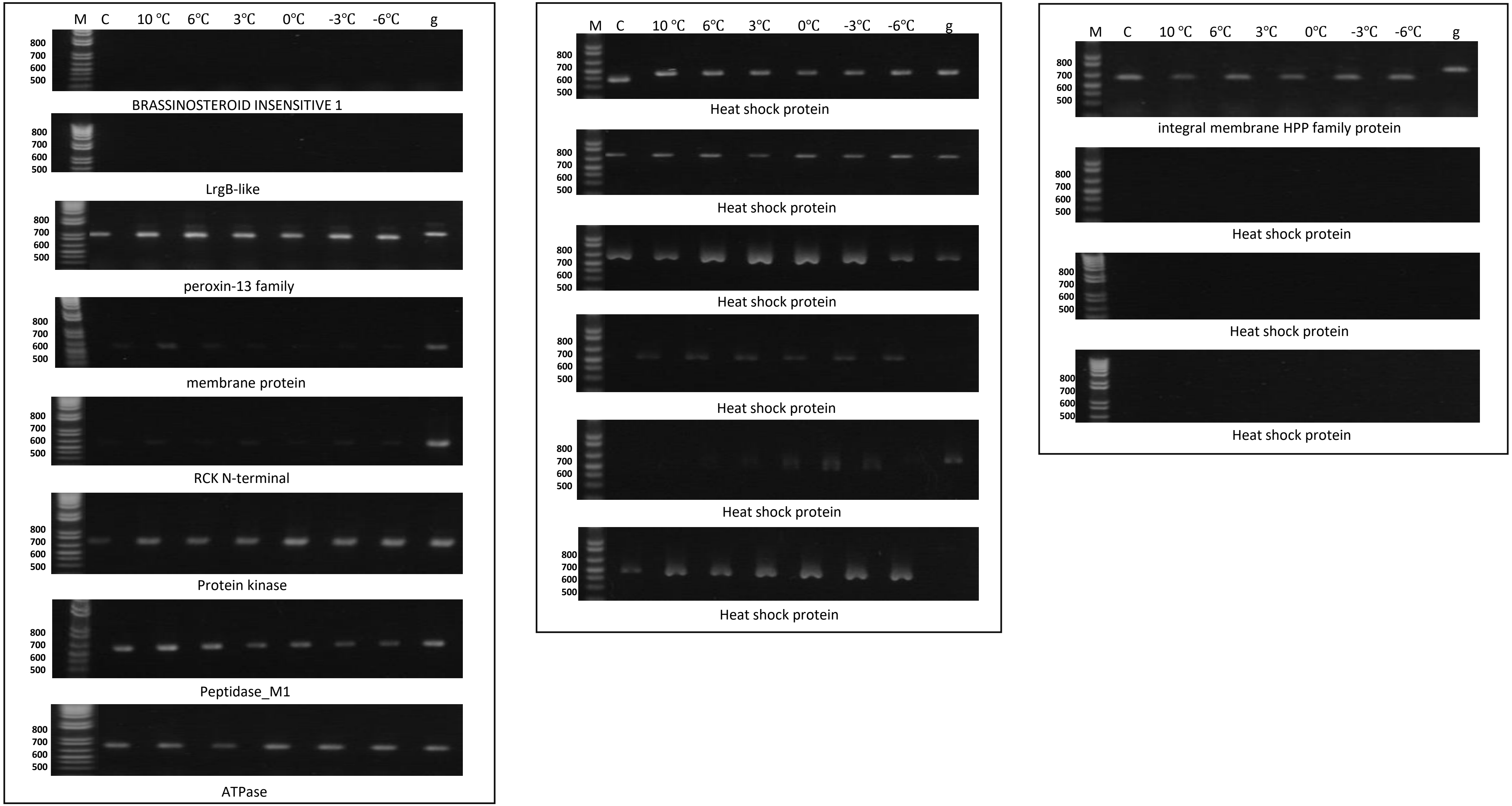

M C 10 °C 6°C 3°C 0°C -3°C -6°C g
800
700
600
500
BRASSINOSTEROID INSENSITIVE 1
LrgB-like
peroxin-13 family
membrane protein
RCK N-terminal
Protein kinase
Peptidase_M1
ATPase
Heat shock protein
Heat shock protein
Heat shock protein
Heat shock protein
Heat shock protein
Heat shock protein
integral membrane HPP family protein
Heat shock protein
Heat shock protein
Heat shock protein

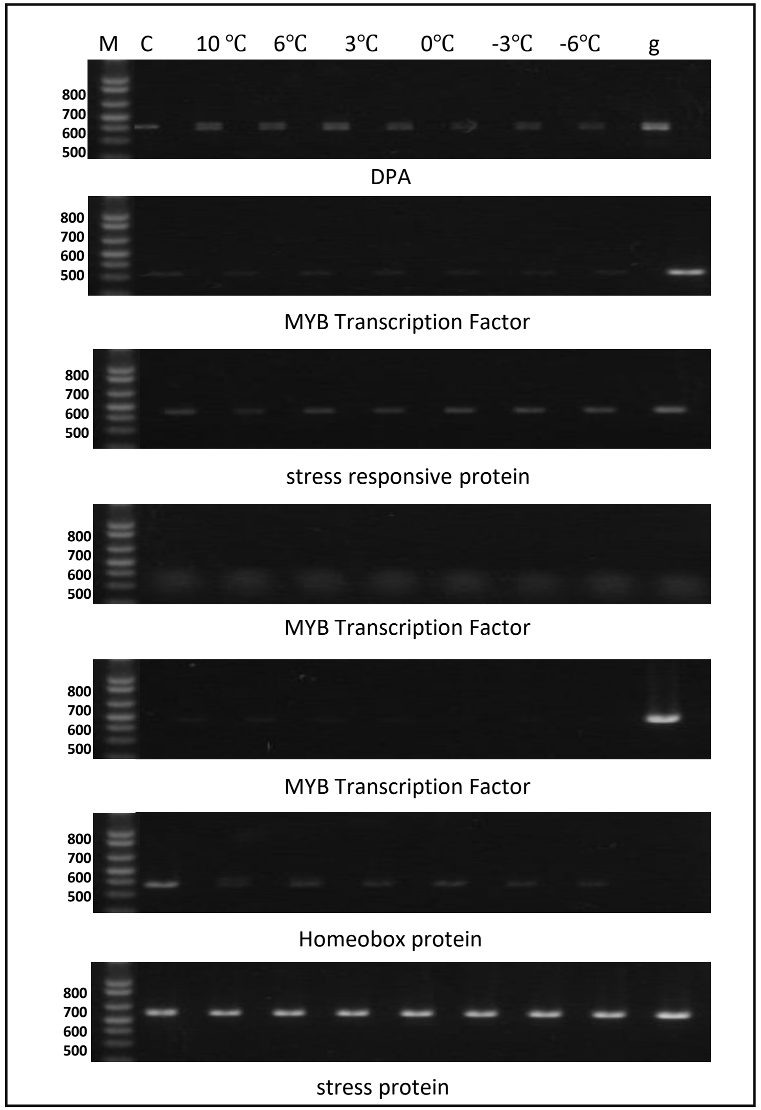
M C 10 ℃ 6℃ 3℃ 0℃ -3℃ -6℃ g
800
700
600
500
DPA
MYB Transcription Factor
stress responsive protein
MYB Transcription Factor
MYB Transcription Factor
Homeobox protein
stress protein

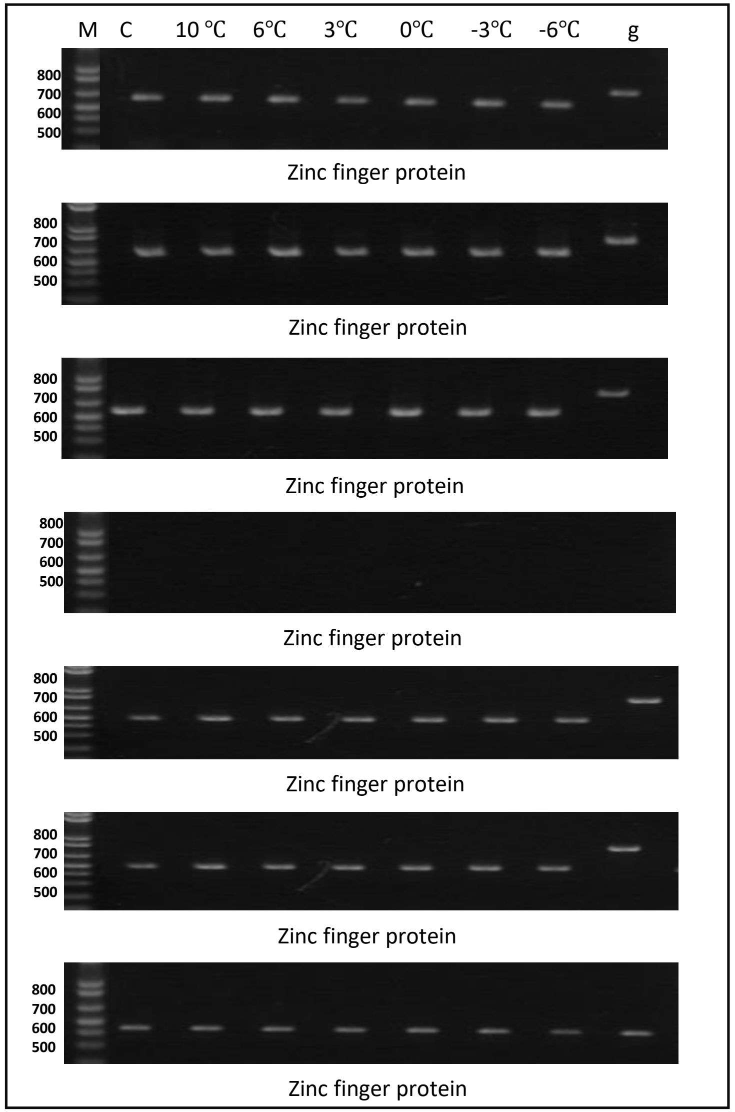
M C 10 ℃ 6℃ 3℃ 0℃ -3℃ -6℃ g
800
700
600
500
Zinc finger protein
Zinc finger protein
Zinc finger protein
Zinc finger protein
Zinc finger protein
Zinc finger protein
Zinc finger protein

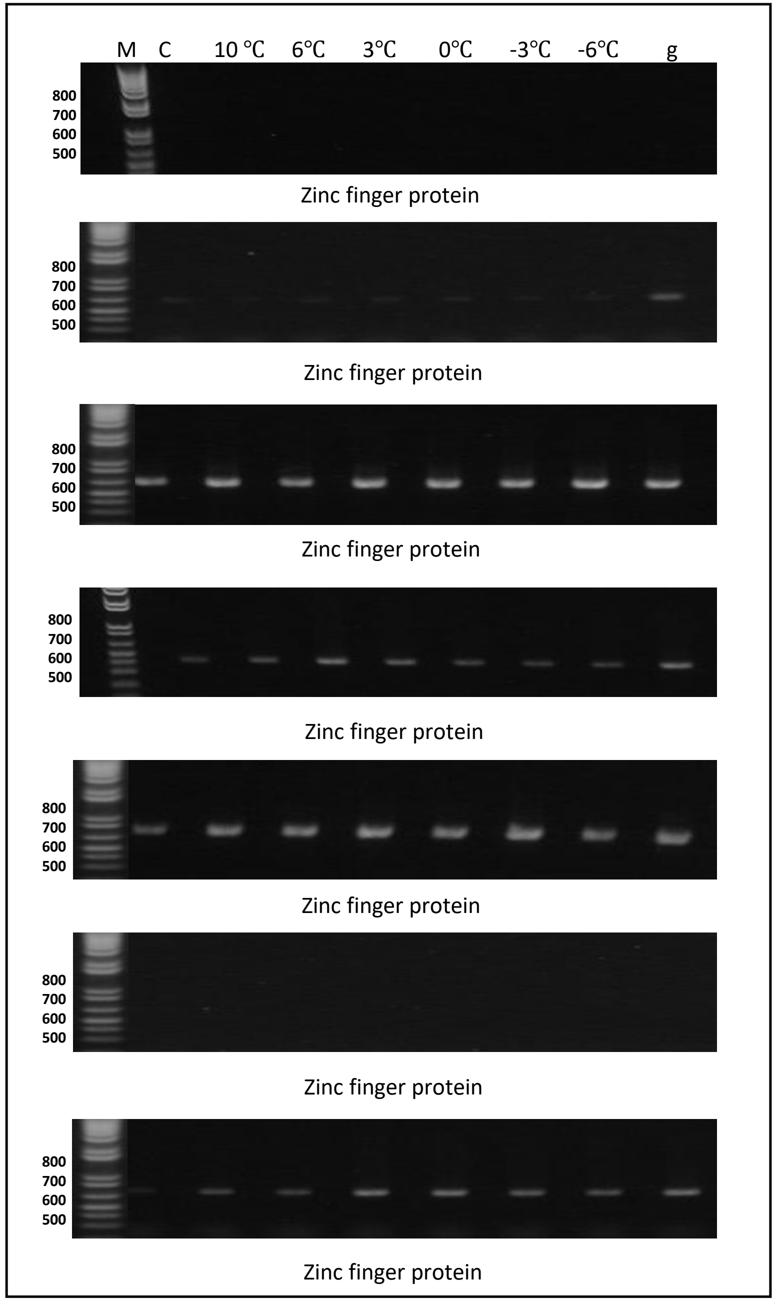
M C 10 ℃ 6℃ 3℃ 0℃ -3℃ -6℃ g
800 700 600 500
Zinc finger protein
800 700 600 500
Zinc finger protein
800 700 600 500
Zinc finger protein
800 700 600 500
Zinc finger protein
800 700 600 500
Zinc finger protein
800 700 600 500
Zinc finger protein
800 700 600 500
Zinc finger protein

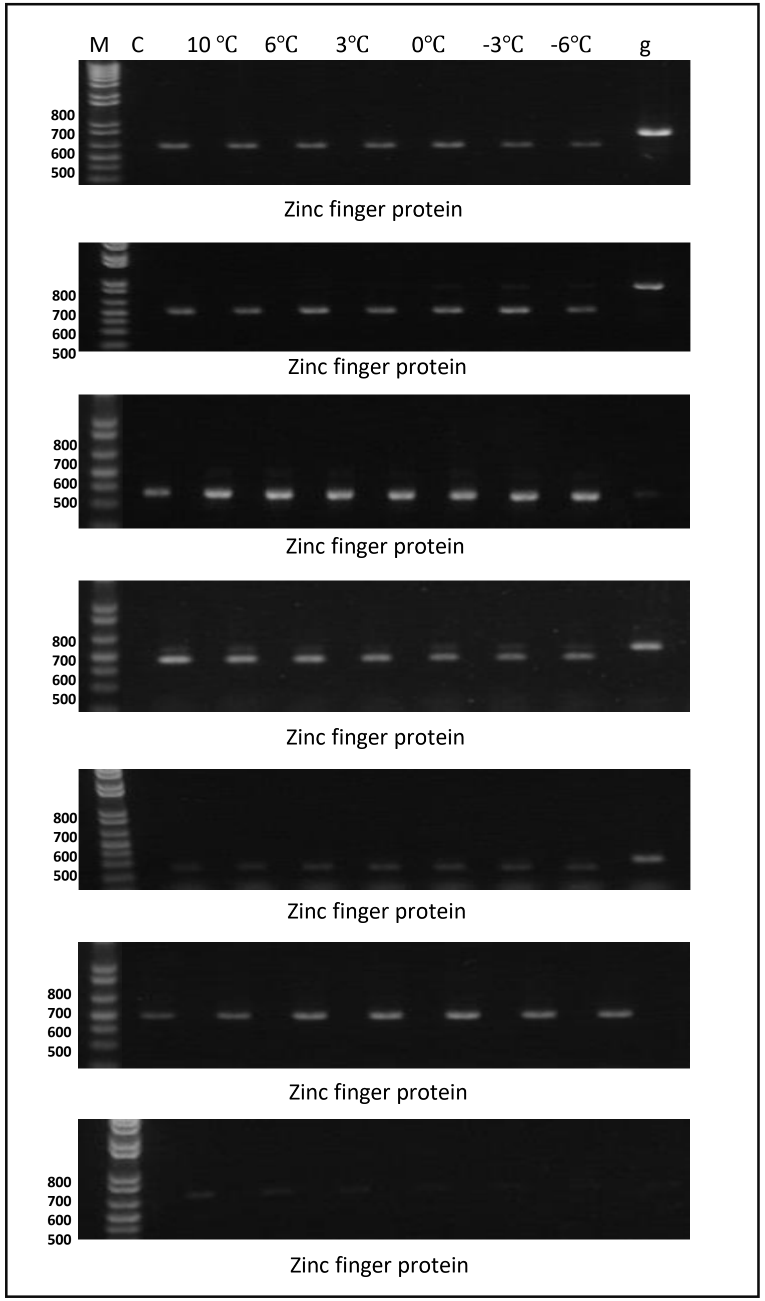
M C 10 ℃ 6℃ 3℃ 0℃ -3℃ -6℃ g
800 700 600 500
Zinc finger protein
800 700 600 500
Zinc finger protein
800 700 600 500
Zinc finger protein
800 700 600 500
Zinc finger protein
800 700 600 500
Zinc finger protein
800 700 600 500
Zinc finger protein
800 700 600 500
Zinc finger protein

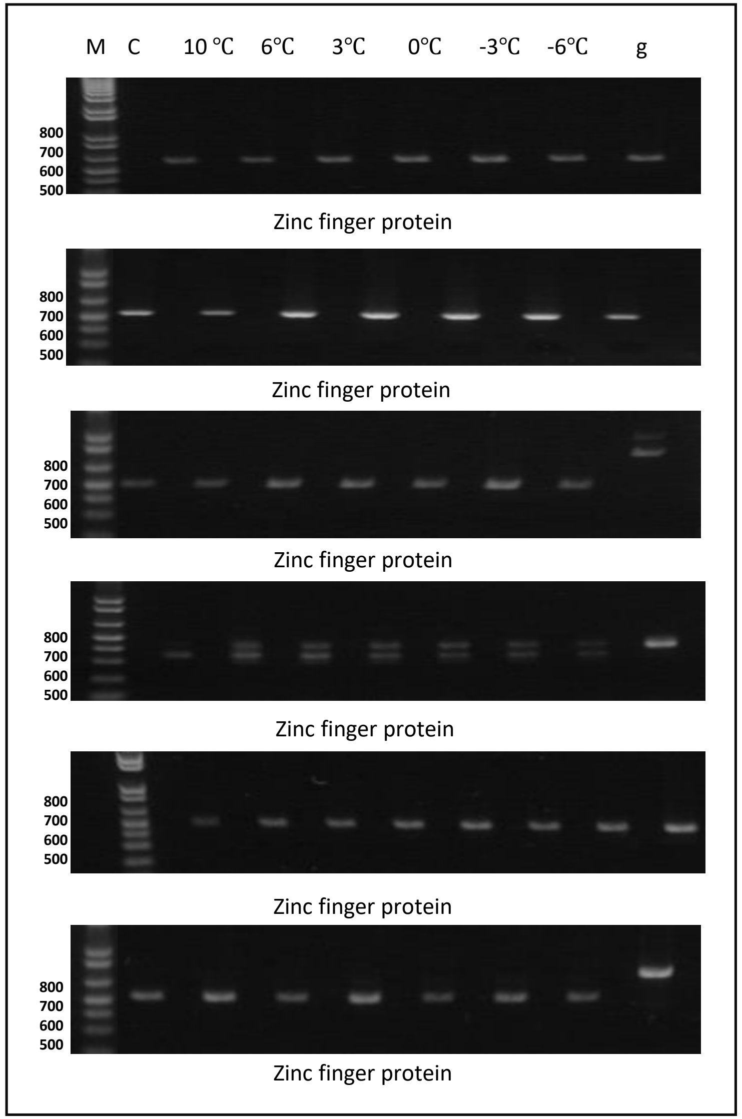
M C 10 ℃ 6℃ 3℃ 0℃ -3℃ -6℃ g
800 700 600 500
Zinc finger protein
800 700 600 500
Zinc finger protein
800 700 600 500
Zinc finger protein
800 700 600 500
Zinc finger protein
800 700 600 500
Zinc finger protein
800 700 600 500
Zinc finger protein
